# A redetermination of the crystal structure of the mannitol complex NH_4_[Mo_2_O_5_(C_6_H_11_O_6_)]·H_2_O: hydrogen-bonding scheme and Hirshfeld surface analysis

**DOI:** 10.1107/S2056989020002935

**Published:** 2020-03-10

**Authors:** Masoud Mirzaei, Morteza Tahmasebi, Joel T. Mague

**Affiliations:** a Department of Chemistry, Ferdowsi University of Mashhad (FUM), Mashhad, PO Box 917751436, Iran; b Department of Chemistry, Tulane University, New Orleans, LA 70118, USA

**Keywords:** crystal structure, mannitol, molybdenum complex, hydrogen bond

## Abstract

The redetermined structure [for the previous study, see: Godfrey & Waters (1975 ▸). *Cryst. Struct. Commun.* **4**, 5–8] of ammonium μ-oxido-μ-[1,5,6-tri­hydroxy­hexane-2,3,4-tris­(olato)]bis­[dioxidomolybdenum(V)] monohydrate, NH_4_[Mo_2_(C_6_H_11_O_6_)O_5_]·H_2_O, was obtained from an attempt to prepare a glutamic acid complex from the [Co_2_Mo_10_H_4_O_38_]^6−^ anion. Subsequent study indicated the complex arose from a substantial impurity of mannitol in the glutamic acid sample used. All hydrogen atoms have been located in the present study and the packing displays N—H⋯O, O—H⋯O and C—H⋯O hydrogen bonds. A Hirshfeld surface analysis was also performed.

## Abstract 2

The redetermined structure of the title compound, [H_4_N][C_6_H_11_Mo_2_O_11_]· H_2_O, was obtained from an attempt to prepare a glutamic acid complex from the [Co_2_Mo_10_H_4_O_38_]^6−^ anion.

## Chemical context   

Over the past few years, there has been considerable inter­est in derivatives of polyoxo- and heteropolyxometallates for both biological and materials applications, particularly where chirality may be conferred by the attachment of chiral ligands (Arefian *et al.*, 2017 ▸; Proust *et al.*, 2012 ▸; Mirzaei *et al.*, 2014 ▸; An *et al.*, 2006 ▸). Recently our group prepared the aspartate complex [Co_2_(C_4_H_6_NO_4_)_2_(γ-Mo_8_O_26_)(H_2_O)_10_]·4H_2_O from (NH_4_)_6_[Co_2_Mo_10_H_4_O_38_], and l-aspartic acid (Tahmasebi *et al.*, 2019 ▸) and have now proceeded to explore the generality of this reaction with other chiral amino acids. We report here on the reaction of the heteropolyoxometallate with l-glutamic acid from which a mannitol complex of molybdenum was obtained as a result of the unexpected presence of a substantial impurity of mannitol in the glutamic acid sample used.
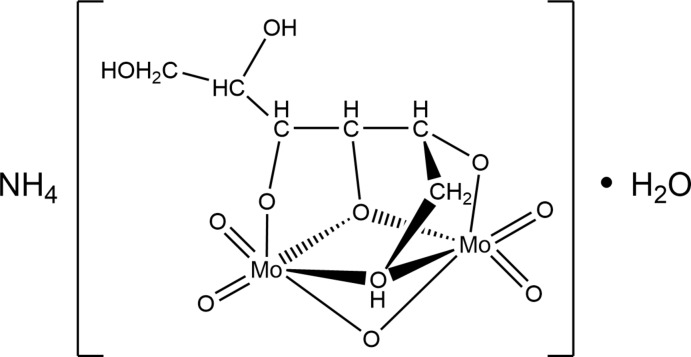


## Structural commentary   

**Figure 1:**
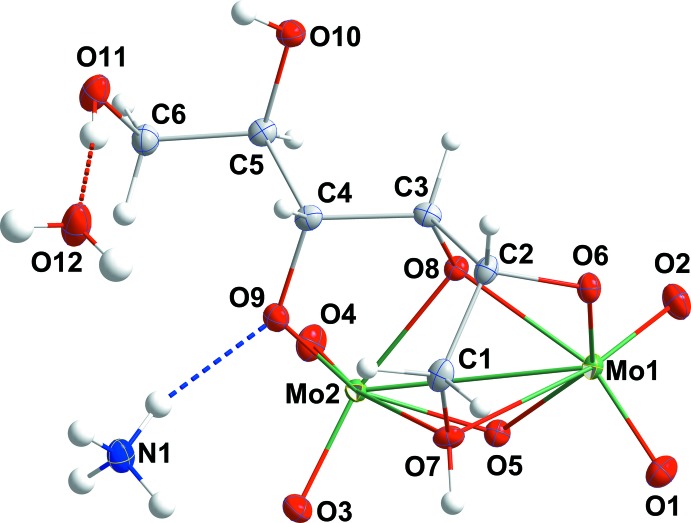
The asymmetric unit with the atom-labeling scheme and 50% probability ellipsoids. The hydrogen bonds from the cation to the anion and from the anion to the water mol­ecule of crystallization are shown by dashed lines.

Instead of the expected complex containing glutamate ligands, the crystals obtained were found to have a unit cell essentially identical to that reported previously for a compound formulated as NH_4_[Mo_2_O_5_(C_6_H_12_O_6_)]·H_2_O (Godfrey & Waters, 1975 ▸) and the structure obtained indicates that it is the same complex. Subsequent to the identification of the product as a mannitol complex, the original sample of glutamic acid was checked by ^1^H and ^13^C NMR spectroscopy and found to contain a significant amount of mannitol as an impurity, thus explaining the formation of the title complex. A comparison of the geometry of the {Mo_2_O_9_} skeleton found in the present study with that in the previous report (Table 1 ▸) indicates the two to be essentially identical, although the present structure, using low-temperature data and more modern instrumentation and software, is of improved precision. A particular feature is that all hydrogen atoms could be located in a difference map and those attached to the oxygen atoms of the mannitol ligand could be refined (although we ultimately chose to fix them in idealized positions because of the presence of heavy metal atoms), making it abundantly clear that three hydroxyl groups on the ligand are deprotonated and also providing a more complete description of the inter­molecular hydrogen-bonding scheme. The terminal Mo=O distances (Table 1 ▸ and Fig. 1 ▸) are short, indicating a degree of multiple bonding while those to O6 and O9 are longer and consistent with single bonds. For the bridging oxygen atoms, O5, O8 and O7, the Mo—O distances for O7 are about the same as for those to O6 and O9, consistent with this atom being a bridging oxide ion. Those to O8 are somewhat longer, as expected for a bridging alkoxide ion, while those to O7 are considerably longer. The previous authors (Godfrey & Waters, 1975 ▸) attributed this ‘*at least in part to stereochemical strain*’ but there is no indication from the relevant bond angles that this is the case. Having located all of the hydrogen atoms, we see that O7 is a hydroxyl group and so would be expected to be less strongly bound to the metal than the anionic oxygen atoms. The Mo1⋯Mo2 separation is 3.1579 (7) Å.

## Supra­molecular features   

**Figure 2:**
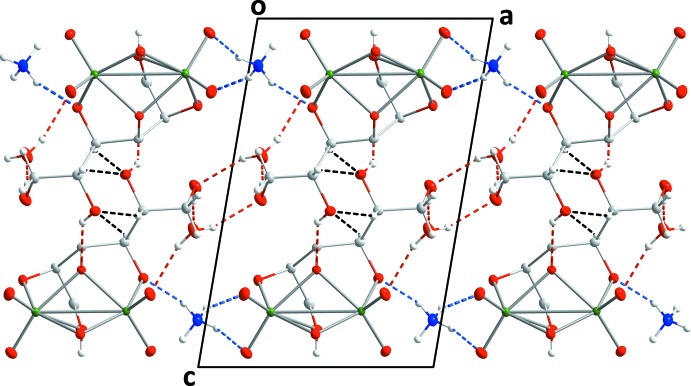
Packing viewed along the *c*-axis direction. O—H⋯O, N—H⋯O and C—H⋯O hydrogen bonds are shown, respectively, by red, blue and black dashed lines

The presence of the ammonium ion, water mol­ecule of crystallization and the remaining hydroxyl groups on the mannitol ligand generates an extensive hydrogen-bonding network in the crystal, which was alluded to in the previous work (Godfrey & Waters, 1975 ▸) but not described. From Table 2 ▸, it may be seen that each ammonium ion connects three adjacent anions through N1—H1*D*⋯O1^i^, N1—H1*E*⋯O2^ii^ and N1—H1*F*⋯O1^iii^ hydrogen bonds [symmetry codes: (i) −1 + *x*, *y*, *z*; (ii) −1 + *x*, −1 + *y*, *z*; (iii) 1 − *x*, −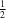 + *y*, −*z*] while each water mol­ecule connects anions by O12—H12*A*⋯O4^v^ and O12—H12*B*⋯O11^vi^ hydrogen bonds [symmetry codes: (v) *x*, −1 + *y*, *z*; (vi) 1 − *x*, −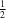 + *y*, 1 − *z*]. The anion at *x*, *y*, *z* is connected to one at (1 − *x*, −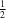 + *y*, −*z*) by an O7—H7⋯O5^iii^ hydrogen bond and to one at (1 − *x*, −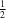 + *y*, 1 − *z*) by an O10—H10⋯O8^iv^ hydrogen bond. Two C—H⋯O hydrogen bonds, one relatively strong and the other weak (Table 2 ▸) complete the inter­molecular inter­actions The result is a structure in which layers of anions, formed by the O—H⋯O and C—H⋯O hydrogen bonds between them, are arranged parallel to the *bc* plane and are connected along the *a*-axis direction by the O—H⋯O and N—H⋯O hydrogen bonds to the cation and the water mol­ecule of crystallization (Figs. 2 ▸ and 3 ▸).

## Database survey   

A search of the Cambridge Crystallographic Database (CSD version 5.41 updated to November 2019; Groom, *et al.*, 2016 ▸) for a triply deprotonated mannitol ion with two Group 6 metals attached found only (NH_4_)[Mo_2_O_5_(C_6_H_12_O_6_)]·H_2_O (XMANMO; Godfrey & Waters, 1975 ▸) and Na[Mo_2_O_5_(C_6_H_12_O_6_)]·2H_2_O (MANMOL10; Hedman, 1977 ▸). From Table 1 ▸, the geometries of the {Mo_2_O_9_} core in all three structures are quite comparable. The packing in MANMOL10 is also quite similar to that seen in the present work, particularly when viewed along the *b*-axis direction although the channel (Fig. 2 ▸) between anions contains sodium cations in place of ammonium cations so there are different hydrogen-bonding inter­actions.

## Hirshfeld surface analysis   

**Figure 4:**
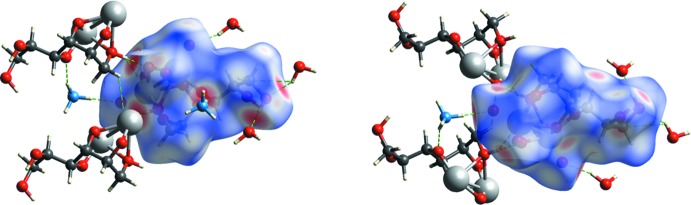
Two views of the Hirshfeld surface for the anion mapped over *d*_norm_ over the range −0.779 to +1.091 arbitrary units with the nearest hydrogen-bonded neighbors added.

**Figure 5:**
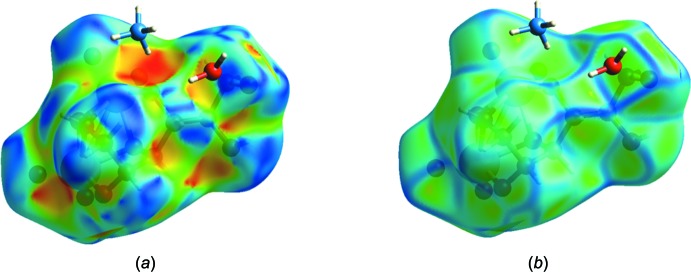
The Hirshfeld surface for the asymmetric unit mapped over (*a*) the shape-index property and (*b*) the curvedness property.

**Figure 6:**
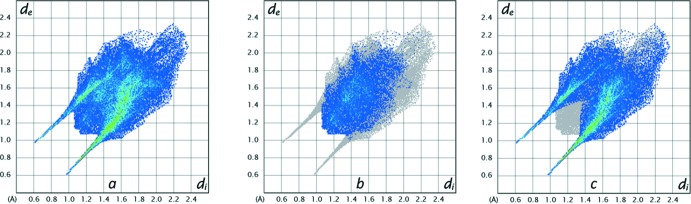
The full two-dimensional fingerprint plot for (*a*) the anion and those delineated into (*b*) H⋯H and (*c*) O—H⋯H—O plus N—H⋯H—O contacts.

The calculation and analysis of the Hirshfeld surface (McKinnon *et al.*, 2007 ▸; Spackman & Jayatilaka, 2009 ▸) can provide information on the presence and directionality of packing inter­actions in a crystal; for example, strong and weak hydrogen bonds and π-stacking and C—H⋯π(ring) inter­actions. The characteristics and appearance of the Hirshfeld surface and related surfaces and fingerprint plots that can be generated with *CrystalExplorer 17* (Turner *et al.*, 2017 ▸) have been fully described (Tan *et al.*, 2019 ▸). Two views of the Hirshfeld surface mapped over *d*_norm_ are shown in Fig. 4 ▸ *a* and Fig. 4 ▸ *b*, which include the entities making the closest contacts as listed in Table 2 ▸. The O—H⋯O and N—H⋯O hydrogen bonds to and within the asymmetric unit are clearly shown by the dark-red circles while the light-red ones indicate weak C—H⋯O inter­actions: these are consistent with the extensive hydrogen-bonding network depicted in Figs. 2 ▸ and 3 ▸. The Hirshfeld surface mapped over shape index (Fig. 5 ▸ *a*) and curvedness (Fig. 5 ▸ *b*) indicate, as expected from the X-ray structure, that the anion is compact with relatively little flat surface exposed to its neighboring ions. Fig. 6 ▸ *a* shows the overall fingerprint plot while Fig. 6 ▸ *b* and 6*c* show delineation into H⋯H, and O—H⋯H—O plus N—H⋯H—O inter­actions, respectively. The former comprises 27.4% of the surface while the latter comprises 66%, again emphasizing the extensive O—H⋯O and N—H⋯O hydrogen bonding present. Of particular note in Fig. 6 ▸ *c* are the two spikes at *d*_e_ + *d*_i_ = 1.56 Å, which is over 1.3 Å less than the sum of the van der Waals radii and consistent with the prevalence of these two types of hydrogen bonding.

## Synthesis and crystallization   

(NH_4_)_6_[Co_2_Mo_10_H_4_O_38_]·7H_2_O (0.29 g, 0.15 mmol) was dissolved in 8 ml of water and 4 ml of ethanol were added, giving a solution pH above 4. Then, 8 ml of an aqueous solution of supposed l-glutamic acid, C_5_H_9_NO_4_ (0.13 g, 0.9 mmol), was added leading to a solution pH of 3.2. The solution was stirred for 2 h and then transferred to a Teflon-lined autoclave (30 ml) and kept at 383 K for 72 h. After the mixture had been cooled slowly to room temperature, it was filtered and with slow evaporation of the solution at room temperature, flat colorless crystals of the title compound were obtained in 73% yield (based on Mo). Subsequent to the identification of the crystals as a mannitol complex, the original sample of glutamic acid was examined by ^1^H and ^13^C NMR and these spectra clearly showed the glutamic acid to be contaminated by a substantial qu­antity of mannitol.

## Refinement details   

Crystal data, data collection and structure refinement details are summarized in Table 3 ▸. H atoms attached to carbon were placed in calculated positions (C—H = 0.99–1.00 Å) while those attached to oxygen and to nitro­gen were placed in locations derived from a difference map, refined for a few cycles to ensure that reasonable displacement parameters could be achieved, and then their coordinates were adjusted to give O—H = 0.87 and N—H = 0.88 Å. All were included as riding contributions with isotropic displacement parameters 1.2–1.5 times those of the parent atoms.

## Supplementary Material

Crystal structure: contains datablock(s) global2, I. DOI: 10.1107/S2056989020002935/hb7879sup1.cif

Structure factors: contains datablock(s) I. DOI: 10.1107/S2056989020002935/hb7879Isup2.hkl

CCDC reference: 1965028

Additional supporting information:  crystallographic information; 3D view; checkCIF report

## Figures and Tables

**Figure 3:**
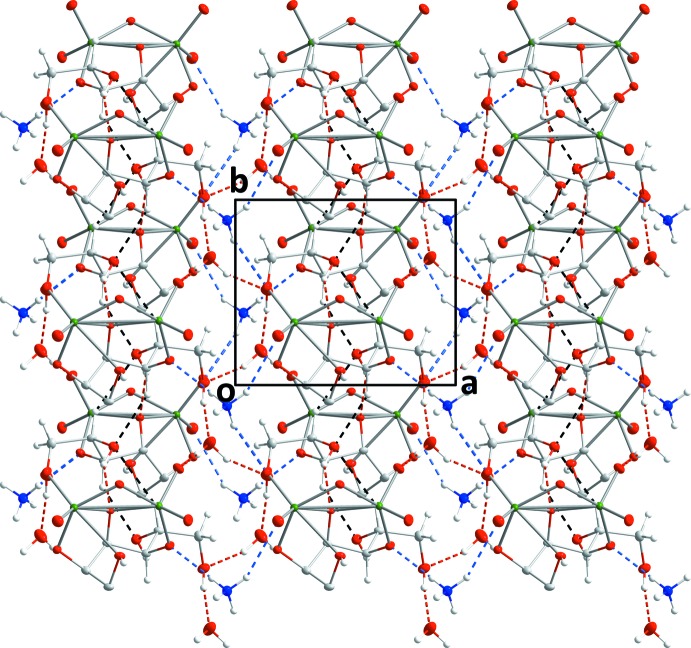
Packing viewed along the *b*-axis direction with inter­molecular hydrogen bonds depicted as in Fig. 2 ▸.

**Table 1:** Comparison of the geometries of the {Mo_2_O_9_} fragment (Å, °)

	This work	XMANMO^*a*^	MANMOL10^*b*^
Mo1⋯Mo2	3.1579 (7)	3.147 (2)	3.1435 (3)
Mo1—O1	1.720 (2)	1.722 (5)	1.727 (3)
Mo1—O2	1.703 (2)	1.679 (5)	1.693 (3)
Mo1—O5	1.937 (2)	1.940 (5)	1.936 (2)
Mo1—O6	1.939 (2)	1.998 (5)	1.931 (2)
Mo1—O7	2.454 (2)	2.469 (5)	2.459 (2)
Mo1—O8	2.1733 (19)	2.175 (5)	2.162 (1)
Mo2—O3	1.713 (2)	1.711 (5)	1.716 (2)
Mo2—O4	1.710 (2)	1.690 (5)	1.703 (2)
Mo2—O5	1.955 (2)	1.952 (5)	1.950 (1)
Mo2—O9	1.941 (2)	1.958 (5)	1.925 (2)
Mo2—O7	2.478 (2)	2.530 (5)	2.489 (2)
Mo2—O8	2.1220 (19)	2.120 (5)	2.113 (1)
			
O1—Mo1—O2	105.18 (12)	107.0 (5)	103.00 (11)
O3—Mo2—O4	106.63 (12)	107.3 (5)	104.19 (11)
O2—Mo1—O5	104.04 (11)	103.6 (5)	105.02 (9)
O4—Mo2—O5	102.25 (11)	102.0 (5)	100.90 (8)
O5—Mo1—O8	72.27 (8)	72.9 (4)	72.33 (5)
O5—Mo2—O8	73.08 (8)	73.9 (4)	73.18 (5)

Notes: (*a*) Godfrey & Waters (1975 ▸); (*b*) Hedman (1977 ▸).

**Table 2:** Hydrogen-bond geometry (Å, °)

*D*—H⋯*A*	*D*—H	H⋯*A*	*D*⋯*A*	*D*—H⋯*A*
N1—H1*D*⋯O1^i^	0.88	2.46	3.272 (4)	154
N1—H1*E*⋯O2^ii^	0.88	2.22	3.066 (4)	162
N1—H1*F*⋯O1^iii^	0.88	1.95	2.831 (4)	177
O7—H7⋯O5^iii^	0.87	1.72	2.589 (3)	178
O10—H10⋯O8^iv^	0.87	2.37	3.137 (3)	148
O12—H12*A*⋯O4^v^	0.87	2.01	2.845 (4)	162
O12—H12*B*⋯O11^vi^	0.87	2.02	2.812 (3)	151
C4—H4⋯O10^iv^	1.00	2.37	3.243 (4)	145
C5—H5⋯O10^vii^	1.00	2.58	3.491 (4)	151

Symmetry codes: (i) 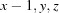; (ii) 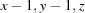; (iii) 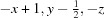; (iv) 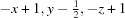; (v) 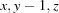; (vi) 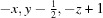; (vii) 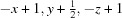.

**Table 3:** Experimental details

Crystal data	
Chemical formula	NH_4_[Mo_2_(C_6_H_11_O_6_)O_5_]·H_2_O
*M*_r_	487.08
Crystal system, space group	Monoclinic, *P*2_1_
Temperature (K)	150
*a*, *b*, *c* (Å)	8.1775 (17), 6.7722 (14), 12.305 (3)
β (°)	99.664 (3)
*V* (Å^3^)	671.8 (2)
*Z*	2
Radiation type	Mo *K*α
μ (mm^−1^)	1.93
Crystal size (mm)	0.28 × 0.17 × 0.07

Data collection	
Diffractometer	Bruker SMART APEX CCD
Absorption correction	Multi-scan (*SADABS*; Krause *et al.*, 2015 ▸)
*T*_min_, *T*_max_	0.61, 0.88
No. of measured, independent and observed [*I* > 2σ(*I*)] reflections	12645, 3598, 3407
*R*_int_	0.024
(sin θ/λ)_max_ (Å^−1^)	0.695

Refinement	
*R*[*F* ^2^ > 2σ(*F* ^2^)], *wR*(*F* ^2^), *S*	0.019, 0.050, 1.05
No. of reflections	3598
No. of parameters	190
No. of restraints	1
H-atom treatment	H-atom parameters constrained
Δρ_max_, Δρ_min_ (e Å^−3^)	0.91, −0.51
Absolute structure	Flack *x* determined using 1454 quotients [(*I* ^+^)−(*I* ^−^)]/[(*I* ^+^)+(*I* ^−^)] (Parsons *et al.*, 2013 ▸).
Absolute structure parameter	−0.026 (16)

Computer programs: *APEX3* and *SAINT* (Bruker, 2016 ▸), *SHELXT* (Sheldrick, 2015*a* ▸), *SHELXL2018/1* (Sheldrick, 2015*b* ▸), *DIAMOND* (Brandenburg & Putz, 2012 ▸) and *SHELXTL* (Sheldrick, 2008 ▸).

## supplementary crystallographic information

### Crystal data

**Table:**

NH_4_[Mo_2_(C_6_H_11_O_6_)O_5_]·H_2_O	*F*(000) = 480
*M**_r_* = 487.08	*D*_x_ = 2.408 Mg m^−^^3^
Monoclinic, *P*2_1_	Mo *K*α radiation, λ = 0.71073 Å
*a* = 8.1775 (17) Å	Cell parameters from 9659 reflections
*b* = 6.7722 (14) Å	θ = 2.5–29.6°
*c* = 12.305 (3) Å	µ = 1.93 mm^−^^1^
β = 99.664 (3)°	*T* = 150 K
*V* = 671.8 (2) Å^3^	Plate, colourless
*Z* = 2	0.28 × 0.17 × 0.07 mm

### Data collection

**Table:**

Bruker SMART APEX CCD diffractometer	3598 independent reflections
Radiation source: fine-focus sealed tube	3407 reflections with *I* > 2σ(*I*)
Graphite monochromator	*R*_int_ = 0.024
Detector resolution: 8.3333 pixels mm^-1^	θ_max_ = 29.6°, θ_min_ = 1.7°
φ and ω scans	*h* = −11→10
Absorption correction: multi-scan (*SADABS*; Krause *et al.*, 2015)	*k* = −9→9
*T*_min_ = 0.61, *T*_max_ = 0.88	*l* = −17→17
12645 measured reflections	

### Refinement

**Table:**

Refinement on *F*^2^	Secondary atom site location: difference Fourier map
Least-squares matrix: full	Hydrogen site location: mixed
*R*[*F*^2^ > 2σ(*F*^2^)] = 0.019	H-atom parameters constrained
*wR*(*F*^2^) = 0.050	*w* = 1/[σ^2^(*F*_o_^2^) + (0.0303*P*)^2^] where *P* = (*F*_o_^2^ + 2*F*_c_^2^)/3
*S* = 1.05	(Δ/σ)_max_ = 0.001
3598 reflections	Δρ_max_ = 0.91 e Å^−^^3^
190 parameters	Δρ_min_ = −0.51 e Å^−^^3^
1 restraint	Absolute structure: Flack *x* determined using 1454 quotients [(*I*^+^)-(*I*^-^)]/[(*I*^+^)+(*I*^-^)] (Parsons *et al.*, 2013).
Primary atom site location: dual	Absolute structure parameter: −0.026 (16)

### Special details

**Table:**

Experimental. The diffraction data were obtained from 3 sets of 400 frames, each of width 0.5° in ω, colllected at φ = 0.00, 90.00 and 180.00° and 2 sets of 800 frames, each of width 0.45° in φ, collected at ω = –30.00 and 210.00°. The scan time was 10 sec/frame.
Geometry. All esds (except the esd in the dihedral angle between two l.s. planes) are estimated using the full covariance matrix. The cell esds are taken into account individually in the estimation of esds in distances, angles and torsion angles; correlations between esds in cell parameters are only used when they are defined by crystal symmetry. An approximate (isotropic) treatment of cell esds is used for estimating esds involving l.s. planes.
Refinement. Refinement of F^2^ against ALL reflections. The weighted R-factor wR and goodness of fit S are based on F^2^, conventional R-factors R are based on F, with F set to zero for negative F^2^. The threshold expression of F^2^ > 2sigma(F^2^) is used only for calculating R-factors(gt) etc. and is not relevant to the choice of reflections for refinement. R-factors based on F^2^ are statistically about twice as large as those based on F, and R- factors based on ALL data will be even larger. H-atoms attached to carbon were placed in calculated positions (C—H = 0.99 - 1.00 Å) while those attached to nitrogen and oxygen were placed in locations derived from a difference map and their coordinates adjusted to give N—H = 0.88 and O—H = 0.87 %A. All were included as riding contributions with isotropic displacement parameters 1.2 - 1.5 times those of the attached atoms.

### Fractional atomic coordinates and isotropic or equivalent isotropic displacement parameters (Å^2^)

**Table:**

	*x*	*y*	*z*	*U*_iso_*/*U*_eq_	
Mo1	0.73216 (3)	0.83802 (4)	0.15779 (2)	0.00976 (7)	
Mo2	0.34681 (3)	0.84715 (3)	0.16065 (2)	0.00929 (7)	
O1	0.8054 (3)	0.7655 (4)	0.04086 (19)	0.0185 (5)	
O2	0.8566 (3)	1.0290 (4)	0.2099 (2)	0.0189 (5)	
O3	0.2139 (3)	0.7705 (4)	0.04573 (19)	0.0168 (5)	
O4	0.2581 (3)	1.0501 (4)	0.2096 (2)	0.0182 (5)	
O5	0.5254 (3)	0.9641 (3)	0.09446 (18)	0.0117 (4)	
O6	0.8084 (3)	0.6119 (3)	0.24906 (19)	0.0127 (4)	
O7	0.5241 (3)	0.5776 (3)	0.10930 (17)	0.0124 (4)	
H7	0.506325	0.541607	0.040453	0.019*	
O8	0.5670 (2)	0.8230 (4)	0.27874 (15)	0.0095 (4)	
O9	0.2980 (3)	0.6327 (3)	0.25489 (19)	0.0131 (5)	
O10	0.4487 (3)	0.6669 (3)	0.55252 (18)	0.0148 (5)	
H10	0.403779	0.570084	0.583505	0.022*	
O11	0.1432 (3)	0.5102 (4)	0.5185 (2)	0.0193 (5)	
H11	0.142714	0.406135	0.477050	0.029*	
C1	0.5738 (4)	0.4105 (5)	0.1814 (3)	0.0137 (6)	
H1A	0.640081	0.315372	0.145831	0.016*	
H1B	0.475537	0.341650	0.200268	0.016*	
C2	0.6776 (4)	0.4998 (5)	0.2839 (2)	0.0123 (6)	
H2	0.725734	0.392308	0.335185	0.015*	
C3	0.5819 (4)	0.6456 (4)	0.3449 (3)	0.0107 (6)	
H3	0.648235	0.674825	0.419035	0.013*	
C4	0.4041 (4)	0.5921 (5)	0.3577 (2)	0.0116 (6)	
H4	0.398270	0.448370	0.375349	0.014*	
C5	0.3477 (4)	0.7141 (4)	0.4496 (3)	0.0119 (6)	
H5	0.365147	0.856715	0.433853	0.014*	
C6	0.1656 (4)	0.6852 (5)	0.4567 (3)	0.0160 (6)	
H6A	0.124332	0.801416	0.492730	0.019*	
H6B	0.100498	0.673866	0.381457	0.019*	
O12	0.1166 (3)	0.1850 (4)	0.3922 (2)	0.0268 (6)	
H12A	0.172118	0.125773	0.347290	0.040*	
H12B	0.039628	0.100843	0.401180	0.040*	
N1	0.0369 (3)	0.3889 (4)	0.1356 (2)	0.0167 (6)	
H1C	0.107045	0.457351	0.183173	0.025*	
H1D	−0.050964	0.461341	0.111363	0.025*	
H1E	0.006546	0.282251	0.167903	0.025*	
H1F	0.085396	0.354261	0.079803	0.025*	

### Atomic displacement parameters (Å^2^)

**Table:**

	*U*^11^	*U*^22^	*U*^33^	*U*^12^	*U*^13^	*U*^23^
Mo1	0.00916 (12)	0.00909 (11)	0.01150 (11)	−0.00040 (13)	0.00308 (8)	0.00066 (13)
Mo2	0.00860 (12)	0.00943 (11)	0.00969 (11)	0.00069 (13)	0.00110 (8)	0.00059 (13)
O1	0.0198 (13)	0.0200 (11)	0.0174 (11)	0.0015 (10)	0.0078 (10)	0.0001 (10)
O2	0.0150 (12)	0.0170 (12)	0.0240 (13)	−0.0051 (10)	0.0007 (10)	0.0000 (10)
O3	0.0140 (12)	0.0201 (11)	0.0154 (11)	−0.0008 (9)	−0.0007 (9)	0.0013 (9)
O4	0.0171 (13)	0.0155 (12)	0.0235 (14)	0.0050 (10)	0.0076 (11)	0.0009 (10)
O5	0.0123 (11)	0.0098 (10)	0.0132 (10)	0.0010 (8)	0.0026 (8)	0.0013 (8)
O6	0.0116 (11)	0.0103 (11)	0.0166 (11)	−0.0011 (9)	0.0034 (9)	0.0005 (9)
O7	0.0177 (12)	0.0097 (10)	0.0100 (10)	0.0007 (9)	0.0026 (8)	−0.0022 (8)
O8	0.0111 (9)	0.0068 (10)	0.0107 (8)	0.0006 (9)	0.0022 (7)	0.0018 (9)
O9	0.0134 (11)	0.0113 (10)	0.0144 (12)	−0.0025 (9)	0.0020 (9)	0.0006 (9)
O10	0.0161 (12)	0.0149 (11)	0.0133 (11)	−0.0009 (9)	0.0020 (9)	0.0005 (8)
O11	0.0227 (13)	0.0148 (11)	0.0227 (12)	−0.0041 (10)	0.0104 (10)	−0.0012 (10)
C1	0.0159 (16)	0.0085 (13)	0.0179 (14)	−0.0005 (11)	0.0060 (12)	0.0010 (11)
C2	0.0128 (15)	0.0108 (14)	0.0142 (14)	0.0011 (11)	0.0051 (12)	0.0027 (11)
C3	0.0128 (15)	0.0077 (13)	0.0111 (13)	0.0011 (11)	0.0002 (12)	0.0015 (11)
C4	0.0144 (15)	0.0093 (13)	0.0113 (14)	−0.0001 (11)	0.0025 (12)	0.0007 (11)
C5	0.0142 (15)	0.0084 (13)	0.0132 (14)	0.0016 (11)	0.0025 (12)	0.0014 (11)
C6	0.0132 (16)	0.0177 (16)	0.0175 (16)	0.0003 (13)	0.0035 (13)	0.0005 (12)
O12	0.0233 (14)	0.0271 (14)	0.0336 (15)	−0.0079 (11)	0.0148 (12)	−0.0136 (12)
N1	0.0136 (14)	0.0153 (16)	0.0215 (13)	0.0004 (9)	0.0043 (11)	−0.0041 (10)

### Geometric parameters (Å, º)

**Table:**

Mo1—O2	1.703 (2)	O11—H11	0.8699
Mo1—O1	1.720 (2)	C1—C2	1.522 (5)
Mo1—O5	1.937 (2)	C1—H1A	0.9900
Mo1—O6	1.939 (2)	C1—H1B	0.9900
Mo1—O8	2.1733 (19)	C2—C3	1.532 (4)
Mo1—O7	2.454 (2)	C2—H2	1.0000
Mo1—Mo2	3.1579 (7)	C3—C4	1.532 (4)
Mo2—O4	1.710 (2)	C3—H3	1.0000
Mo2—O3	1.713 (2)	C4—C5	1.532 (4)
Mo2—O9	1.941 (2)	C4—H4	1.0000
Mo2—O5	1.955 (2)	C5—C6	1.519 (4)
Mo2—O8	2.1220 (19)	C5—H5	1.0000
Mo2—O7	2.478 (2)	C6—H6A	0.9900
O6—C2	1.435 (4)	C6—H6B	0.9900
O7—C1	1.453 (4)	O12—H12A	0.8700
O7—H7	0.8700	O12—H12B	0.8699
O8—C3	1.444 (4)	N1—H1C	0.8800
O9—C4	1.436 (4)	N1—H1D	0.8798
O10—C5	1.427 (4)	N1—H1E	0.8800
O10—H10	0.8700	N1—H1F	0.8800
O11—C6	1.437 (4)		
			
O2—Mo1—O1	105.18 (12)	C3—O8—Mo2	115.55 (17)
O2—Mo1—O5	104.04 (11)	C3—O8—Mo1	114.93 (17)
O1—Mo1—O5	101.08 (10)	Mo2—O8—Mo1	94.64 (7)
O2—Mo1—O6	105.54 (11)	C4—O9—Mo2	121.01 (19)
O1—Mo1—O6	97.81 (11)	C5—O10—H10	109.5
O5—Mo1—O6	139.04 (9)	C6—O11—H11	110.2
O2—Mo1—O8	100.26 (11)	O7—C1—C2	104.9 (2)
O1—Mo1—O8	154.56 (10)	O7—C1—H1A	110.8
O5—Mo1—O8	72.27 (8)	C2—C1—H1A	110.8
O6—Mo1—O8	75.09 (9)	O7—C1—H1B	110.8
O2—Mo1—O7	169.57 (10)	C2—C1—H1B	110.8
O1—Mo1—O7	85.19 (10)	H1A—C1—H1B	108.8
O5—Mo1—O7	72.31 (8)	O6—C2—C1	107.6 (2)
O6—Mo1—O7	73.48 (9)	O6—C2—C3	105.8 (2)
O8—Mo1—O7	69.37 (8)	C1—C2—C3	113.7 (3)
O2—Mo1—Mo2	121.00 (9)	O6—C2—H2	109.8
O1—Mo1—Mo2	120.21 (8)	C1—C2—H2	109.8
O5—Mo1—Mo2	35.97 (6)	C3—C2—H2	109.8
O6—Mo1—Mo2	103.50 (7)	O8—C3—C2	105.2 (2)
O8—Mo1—Mo2	42.05 (5)	O8—C3—C4	105.3 (2)
O7—Mo1—Mo2	50.52 (5)	C2—C3—C4	118.0 (3)
O4—Mo2—O3	106.63 (12)	O8—C3—H3	109.3
O4—Mo2—O9	104.41 (11)	C2—C3—H3	109.3
O3—Mo2—O9	95.78 (11)	C4—C3—H3	109.3
O4—Mo2—O5	102.25 (11)	O9—C4—C5	109.6 (2)
O3—Mo2—O5	101.17 (10)	O9—C4—C3	107.9 (2)
O9—Mo2—O5	142.62 (10)	C5—C4—C3	110.9 (3)
O4—Mo2—O8	100.51 (11)	O9—C4—H4	109.5
O3—Mo2—O8	152.85 (11)	C5—C4—H4	109.5
O9—Mo2—O8	76.66 (9)	C3—C4—H4	109.5
O5—Mo2—O8	73.08 (8)	O10—C5—C6	110.3 (3)
O4—Mo2—O7	169.36 (10)	O10—C5—C4	109.5 (2)
O3—Mo2—O7	83.30 (10)	C6—C5—C4	113.1 (3)
O9—Mo2—O7	77.80 (9)	O10—C5—H5	107.9
O5—Mo2—O7	71.48 (8)	C6—C5—H5	107.9
O8—Mo2—O7	69.65 (8)	C4—C5—H5	107.9
O4—Mo2—Mo1	120.20 (9)	O11—C6—C5	110.5 (3)
O3—Mo2—Mo1	118.74 (8)	O11—C6—H6A	109.6
O9—Mo2—Mo1	107.31 (7)	C5—C6—H6A	109.6
O5—Mo2—Mo1	35.58 (6)	O11—C6—H6B	109.6
O8—Mo2—Mo1	43.31 (5)	C5—C6—H6B	109.6
O7—Mo2—Mo1	49.85 (5)	H6A—C6—H6B	108.1
Mo1—O5—Mo2	108.45 (10)	H12A—O12—H12B	104.1
C2—O6—Mo1	114.04 (18)	H1C—N1—H1D	109.6
C1—O7—Mo1	107.42 (18)	H1C—N1—H1E	109.5
C1—O7—Mo2	122.26 (18)	H1D—N1—H1E	109.5
Mo1—O7—Mo2	79.63 (7)	H1C—N1—H1F	109.3
C1—O7—H7	111.1	H1D—N1—H1F	109.5
Mo1—O7—H7	115.7	H1E—N1—H1F	109.4
Mo2—O7—H7	116.5		
			
Mo1—O7—C1—C2	25.7 (3)	C1—C2—C3—C4	41.6 (4)
Mo2—O7—C1—C2	−62.9 (3)	Mo2—O9—C4—C5	88.7 (3)
Mo1—O6—C2—C1	64.9 (3)	Mo2—O9—C4—C3	−32.1 (3)
Mo1—O6—C2—C3	−57.1 (3)	O8—C3—C4—O9	38.6 (3)
O7—C1—C2—O6	−55.2 (3)	C2—C3—C4—O9	−78.3 (3)
O7—C1—C2—C3	61.7 (3)	O8—C3—C4—C5	−81.4 (3)
Mo2—O8—C3—C2	93.0 (2)	C2—C3—C4—C5	161.7 (3)
Mo1—O8—C3—C2	−15.7 (3)	O9—C4—C5—O10	177.4 (2)
Mo2—O8—C3—C4	−32.3 (3)	C3—C4—C5—O10	−63.6 (3)
Mo1—O8—C3—C4	−141.03 (18)	O9—C4—C5—C6	54.0 (3)
O6—C2—C3—O8	42.6 (3)	C3—C4—C5—C6	173.0 (3)
C1—C2—C3—O8	−75.3 (3)	O10—C5—C6—O11	−40.3 (3)
O6—C2—C3—C4	159.6 (3)	C4—C5—C6—O11	82.6 (3)

### Hydrogen-bond geometry (Å, º)

**Table:**

*D*—H···*A*	*D*—H	H···*A*	*D*···*A*	*D*—H···*A*
N1—H1*D*···O1^i^	0.88	2.46	3.272 (4)	154
N1—H1*E*···O2^ii^	0.88	2.22	3.066 (4)	162
N1—H1*F*···O1^iii^	0.88	1.95	2.831 (4)	177
O7—H7···O5^iii^	0.87	1.72	2.589 (3)	178
O10—H10···O8^iv^	0.87	2.37	3.137 (3)	148
O12—H12*A*···O4^v^	0.87	2.01	2.845 (4)	162
O12—H12*B*···O11^vi^	0.87	2.02	2.812 (3)	151
C4—H4···O10^iv^	1.00	2.37	3.243 (4)	145
C5—H5···O10^vii^	1.00	2.58	3.491 (4)	151

Symmetry codes: (i) *x*−1, *y*, *z*; (ii) *x*−1, *y*−1, *z*; (iii) −*x*+1, *y*−1/2, −*z*; (iv) −*x*+1, *y*−1/2, −*z*+1; (v) *x*, *y*−1, *z*; (vi) −*x*, *y*−1/2, −*z*+1; (vii) −*x*+1, *y*+1/2, −*z*+1.
